# Prospects of Zinc Supplementation in Autism Spectrum Disorders and Shankopathies Such as Phelan McDermid Syndrome

**DOI:** 10.3389/fnsyn.2018.00011

**Published:** 2018-05-23

**Authors:** Simone Hagmeyer, Ann Katrin Sauer, Andreas M. Grabrucker

**Affiliations:** ^1^Institute for Anatomy and Cell Biology, Ulm University, Ulm, Germany; ^2^WG Molecular Analysis of Synaptopathies, Department of Neurology, Neurocenter of Ulm University, Ulm, Germany; ^3^Department of Biological Sciences, University of Limerick, Limerick, Ireland; ^4^Bernal Institute, University of Limerick, Limerick, Ireland; ^5^Health Research Institute (HRI), University of Limerick, Limerick, Ireland

**Keywords:** Zn, autism, ASD, synapse, trace metal, 22q13, 22q13.3

## Abstract

The loss of one copy of SHANK3 (SH3 and multiple ankyrin repeat domains 3) in humans highly contributes to Phelan McDermid syndrome (PMDS). In addition, SHANK3 was identified as a major autism candidate gene. Interestingly, the protein encoded by the SHANK3 gene is regulated by zinc. While zinc deficiency depletes synaptic pools of Shank3, increased zinc levels were shown to promote synaptic scaffold formation. Therefore, the hypothesis arises that patients with PMDS and Autism caused by Shankopathies, having one intact copy of SHANK3 left, may benefit from zinc supplementation, as elevated zinc may drive remaining Shank3 into the post-synaptic density (PSD) and may additional recruit Shank2, a second zinc-dependent member of the SHANK gene family. Further, elevated synaptic zinc levels may modulate E/I ratios affecting other synaptic components such as NMDARs. However, several factors need to be considered in relation to zinc supplementation such as the role of Shank3 in the gastrointestinal (GI) system—the location of zinc absorption in humans. Therefore, here, we briefly discuss the prospect and impediments of zinc supplementation in disorders affecting Shank3 such as PMDS and propose a model for most efficacious supplementation.

## Introduction

### Shank3 and PMDS

Phelan McDermid syndrome (PMDS, also 22q13 deletion syndrome or 22q13.3 deletion syndrome) is classified as a syndromic form of autism due to a majority of patients falling on the autism spectrum, displaying autistic or autism-like behavioral traits, caused by a 22q13.3 deletion that includes the SHANK3 gene. Patients otherwise present with minor facial dysmorphic features, global developmental delay, mental retardation, as well as absent or delayed language acquisition. In addition, ADHD, seizures and gastrointestinal (GI) disorders are common medical comorbid conditions (Wong et al., 1997; Bonaglia et al., 2001; Phelan and McDermid, 2012; Kolevzon et al., 2014; Pfaender et al., 2017).

Heterozygous loss of SHANK3 seems to be a major factor contributing to the pathology of PMDS. In addition, SHANK3 is major autism candidate gene (Leblond et al., 2014). In several patients with autism, deletions, nonsense, missense and splice site mutations have been found that affect the function of one SHANK3 allele (Durand et al., 2007; Gauthier et al., 2009). Proteins of the SHANK (also known as Proline-rich synapse-associated protein ProSAP) family are major scaffold proteins within the post-synaptic density (PSD) of excitatory synapses (Boeckers et al., 2002). A mouse model for PMDS reflecting autistic traits seen in human patients has been published (Bozdagi et al., 2010) and several homozygous Shank3 mutant animals were reported to display autism-like phenotypes including impaired social behavior and ultrasonic vocalizations, repetitive behavior, anxiety and learning and memory problems (Peça et al., 2011; Wang et al., 2011; Schmeisser et al., 2012).

Interestingly, while pharmacological approaches using IGF-1 and CDPPB, a mGluR5 positive allosteric modulator, were partly successful in restoring function in Shank3 deletion model systems (Bozdagi et al., 2010; Verpelli et al., 2011; Wang et al., 2016; Vicidomini et al., 2017), the regulation of Shank2 and Shank3 by zinc may be another promising approach to rescue Shank3 function.

### Zinc and Shank3

The heterozygous loss of the SHANK3 gene results in reduced Shank3 protein levels in PMDS patients. In mutant mice that lack the SHANK3 gene and may function as an animal model for PMDS, a comparable decrease in Shank3 protein levels can be monitored (Peça et al., 2011; Wang et al., 2011; Schmeisser et al., 2012). Intriguingly, a similar reduction in Shank3 protein levels was also observed in animals exposed to a mild zinc deficiency during their embryonic development and in primary hippocampal neurons cultured under zinc deficient conditions (Grabrucker et al., 2011, 2014).

Isoforms of Shank3 and the SHANK family member Shank2 that contain the C-terminal SAM domain directly bind zinc (Baron et al., 2006; Gundelfinger et al., 2006; Grabrucker et al., 2011). Additionally, *in vitro* studies showed that the SAM domain of Shank3 is responsible for its synaptic localization (Boeckers et al., 2005) and oligomerization at the postsynaptic density of glutamatergic synapses (Naisbitt et al., 1999; Baron et al., 2006). The recruitment and multimerization of Shank3 at the PSD is a crucial step in the processes of synapse development and maturation (Grabrucker et al., 2009) and was shown to be highly zinc dependent (Gundelfinger et al., 2006; Grabrucker et al., 2011; Tao-Cheng et al., 2016). Experiments in primary hippocampal neuronal cultures showed that the synaptic localization and protein concentration of Shank3 and Shank2 are highly responsive to alterations in neuronal zinc homeostasis (Grabrucker et al., 2014). In ultrastructural analyses, an increased recruitment of Shank3 to the PSD upon zinc supplementation or stimulation was observed (Tao-Cheng et al., 2016). In addition to that, the presence of zinc was crucial to maintain the augmented Shank3 label intensity at the PSD (Tao-Cheng et al., 2016), and increased the number of synaptic contacts with a “mature” PSD (Grabrucker et al., 2011). On the contrary, an increased number of synapses without a prominent PSD was reported under zinc deficient conditions (Grabrucker et al., 2011), but also Shank deficient conditions. Furthermore, the reduction of neuronal zinc levels by exposure to the highly potent zinc chelators CaEDTA and TPEN resulted in a significant reduction in the number of Shank3 immunoreactive puncta per dendritic length as well as in their fluorescence intensity (Grabrucker et al., 2011, 2014). Additionally, a similar reduction of Shank3 protein levels at synapses was detected by western blot analyses (Grabrucker et al., 2011, 2014). As a consequence of zinc depletion, Shank3 is predominantly diffusely localized in dendritic localizations (Grabrucker et al., 2011) and might enter an inactive state there (Arons et al., 2016) indicating that zinc is crucial for stabilizing Shank3 at the postsynaptic site (Grabrucker et al., 2011; Arons et al., 2016).

Interestingly, the supplementation of primary hippocampal neurons with zinc chloride increased Shank3 immunofluorescence levels and therefore the concentration of Shank3 proteins at synapses (Grabrucker et al., 2011, 2014). In line with the described findings obtained from *in vitro* experiments, prenatal zinc deficiency in mice was found to tremendously affect synaptic Shank3 (Grabrucker et al., 2014). Prenatal zinc deficient pups that were nursed by zinc deficient mothers showed a significant reduction in brain zinc and Shank3 levels. A loss of synaptic Shank3 comparable to that observed in Shank3 knock-out mice was detected in immunohistochemical and biochemical analyses of prenatal zinc deficient animals (Grabrucker et al., 2014). Again, a redistribution of Shank3 from the synaptic site to the cytoplasm was reported (Grabrucker et al., 2014). However, the reduction in zinc levels and the concomitant lack of Shank3 was fully rescued by cross-fostering prenatal zinc deficient pups by mothers fed a zinc adequate control diet indicating that zinc supplementation is sufficient to restore previously diminished Shank3 levels *in vivo* (Grabrucker et al., 2014).

Taken together, reduced Shank3 protein levels comparable to those observed in models of PMDS can be caused by the depletion of zinc emphasizing the strong regulatory effect of zinc on synaptic Shank3. On the other hand, remaining Shank3 protein, and possibly in addition Shank2 proteins, can be recruited to synapses by increasing zinc levels. Therefore, we hypothesize that supplementation with zinc may rescue the loss of Shank3 in PMDS and through this modify the resulting phenotype. It was shown that re-establishing Shank3 levels after birth can ameliorate autism-like symptoms in mice (Mei et al., 2016). However, performing zinc supplementation in Shank3 deficient conditions *in vivo* may face challenges given the reported role of Shank3 in the GI system.

### Shank3 and Zinc Transporters

Aside from GI problems, zinc deficiency was shown to be highly prevalent in individuals with PMDS (Grabrucker et al., 2014; Pfaender and Grabrucker, 2014; Pfaender et al., 2017) and ASD (Yasuda et al., 2011; Arora et al., 2017). With the GI system taking center stage in the maintenance of zinc homeostasis, regulation of zinc levels within the body occurs with help of various proteins including members of the Zinc Transporter (ZnT) and Zrt-and Irt-like proteins (ZIP) superfamily of zinc transporters, and metallothioneins (MT; Fukada et al., 2011; Zhao et al., 2014). In humans, dietary zinc uptake primarily occurs within small intestinal enterocytes (Krebs, 2000; Wang and Zhou, 2010) with ZnT and ZIP transporter moving zinc across cellular membranes and into organelles (Hershfinkel, 2005). Especially, Zip2 and Zip4 proteins act as key players of zinc absorption in enterocytes. Not surprisingly, mutated ZIP4 leads to severe impairments in zinc uptake as seen in the autosomal recessive inherited disorder *Acrodermatitis enteropathica (AE)*. AE patients require lifelong zinc substitution in very large doses as treatment (around 200 mg daily instead of the required daily intake in healthy individuals of 15 mg; Maverakis et al., 2007; Andrews, 2008; Pfaender et al., 2017).

Besides its various other functions in the immune system and during brain development, zinc also plays a vital role in the developing GI system and effects gut morphology (Vela et al., 2015). While current research reports high prevalence of GI disorders and zinc deficiency in neurodevelopmental disorders such as non-syndromic and syndromic ASD, it might also have provided a potential link between these dysfunctions (Vela et al., 2015; Pfaender et al., 2017).

Pfaender et al. (2017) showed that besides its function as postsynaptic scaffolding protein in the brain, Shank3 can additionally be found in human and murine enterocytes in the GI tract. In the same study, analysis of key regulators of trace metal homeostasis revealed significant alterations of ZIP2 and ZIP4 expression on mRNA and protein level in enterocytes generated from PMDS patient derived induced pluripotent stem cells (hiPSC) compared to healthy controls. More so, ZIP2 and ZIP4 expression levels in enterocytes seemed to be dependent on Shank3 protein levels, which was confirmed by overexpression and knockdown experiments in the intestinal Caco-2 cell-line model. Co-immunofluorescence of Shank3 and Zip4 in hiPSC derived enterocytes and co-immunoprecipitation experiments of Shank3 protein using wildtype mouse intestinal epithelial lysate and human enterocyte cell lysates shows that Shank3 proteins seem capable of forming a protein complex with Zip4 and to a lesser extent with Zip2 (Pfaender et al., 2017).

Given that Shank3 levels both *in vitro* and *in vivo* regulate zinc transporter expression, a possible explanation for high incidence rates of zinc deficiency in PMDS can be found. However, the loss of zinc transporters in the GI tract may present a significant challenge for zinc supplementation and similar to AE patients, high levels and daily lifelong supplementation may be needed. Therefore, supplementation with commercially available supplements and dosages reflecting the required daily intake of healthy individuals may not suffice. However, chronic high levels of zinc intake that are not toxic *per se* may have secondary effects for example on copper levels and may be a challenge especially for children with PMDS.

## Perspectives

### ZnAAs as Effective Zinc Supplement in PMDS

In the case of a diet low in zinc, Zip4 at the plasma membrane of enterocytes is upregulated to maximize absorption of zinc (Dufner-Beattie et al., 2004). However, this mechanism seems impaired upon Shank3 depletion (Pfaender et al., 2017). Using novel types of zinc supplements, it is possible to utilize alternative routes of zinc uptake. Recently, we have shown that zinc amino acid complexes (ZnAAs) may provide such alternative absorption/transport pathway opportunity (Sauer et al., 2017). Due to the overall amino acid-like structure, ZnAAs based supplements are taken up by amino acid transporters. For example, using hiPSC from AE patients, we could show that while an inorganic zinc supplement such as ZnCl_2_ performed significantly worse in AE patient cells with non-functional Zip4 compared to control cells, ZnAAs were able to deliver zinc into enterocytes effectively and without significant difference between AE and healthy cells (Sauer et al., 2017). The chemistry of ZnAAs that may be comprised of different amino acids such as glutamate, methionine and lysine, among others, in the form of ZnGlu, ZnMet, ZnLys, allows for release of the zinc (once inside cells or the systemic circulation) whereby zinc will be able to participate in its physiological functions such as the regulation of Shank2 and Shank3 proteins at synapses.

**Figure 1:**
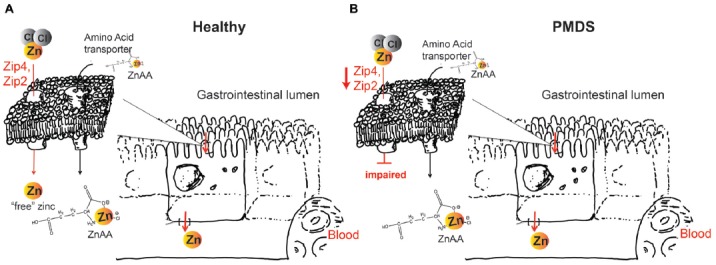
**(A)** Zinc supplements based on zinc salts such as ZnCl_2_ or ZnO will dissociate in solution. In healthy individuals, zinc importers Zip4 and Zip2 take up this “free” (aqueous) zinc ions into enterocytes, which subsequently release zinc on the basolateral side into the blood circulation. Zinc amino acid compounds (ZnAA) are relatively stable and are taken up by amino acid transporters. **(B)** In Phelan McDermid syndrome (PMDS) patients, Zip4 and Zip2 may be affected. Thus, uptake of inorganic zinc supplements may be impaired. In contrast, due to the amino acid-like structure, ZnAAs can still be taken up by amino acid transporters possibly providing more efficient supplementation in PMDS patients.

Therefore, we propose that although supplementation of trace minerals in humans or animals can be performed using different types of supplements, the most suited supplement should be selected, considering the special GI physiology under Shank3 depleted conditions such as PMDS. With a significantly higher demand than that of trace minerals, amino acids will have over a thousand-fold more transporters than any trace mineral. Thus, a selection of ZnAAs for supplementation in PMDS might secure the highest efficacy of zinc supplementation (Figure 1). ZnAAs are currently available on the market as mineral supplement for animals, where they are safe and effective and should be explored in human studies in future. In contrast, using inorganic zinc supplements might need high dosages and long supplementation times until effects might be measurable in Shank3 mouse models and human patients.

### Benefits of Zinc Supplementation in PMDS and ASD—A Hypothesis

**Figure 2:**
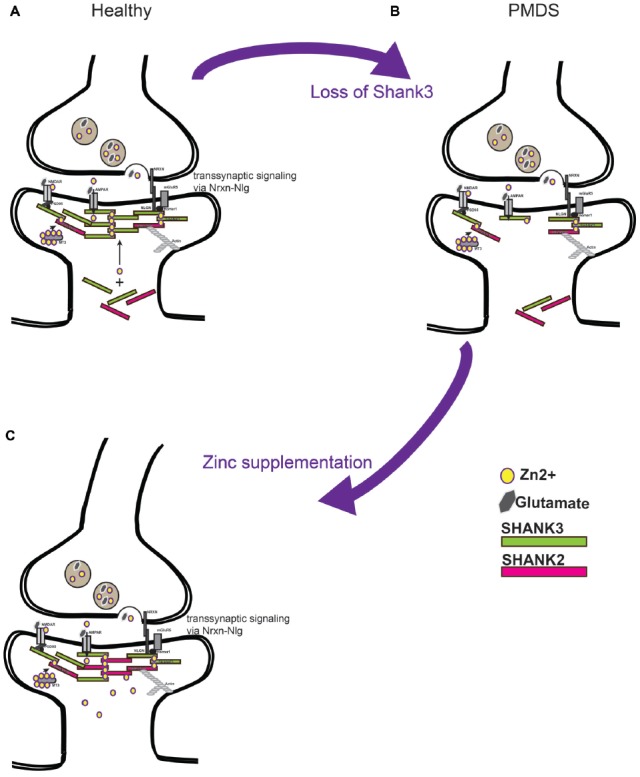
**(A)** Current models predict a soluble pool of Shank proteins at the post-synapse, as well as a post-synaptic density (PSD) bound pool recruited to the PSD by zinc binding. PSD bound Shank scaffolds proteins link receptors at the membrane to the actin cytoskeleton. Further, by binding with Neuroligin (Nlg) and Neurexin (Nrxn) complexes, the level of Shank3 at the PSD provides a transsynaptic signal to the pre-synapse to coordinate synaptic plasticity of both parts of the synapse. **(B)** Loss of Shank3 proteins at the synapse may destabilize synapses and prevent their maturation or formation. Heterozygous deletion or mutation of Shank3 in humans leaves one copy of the gene intact that produces proteins. **(C)** Increasing zinc levels by zinc supplementation might restore Shank3 levels by recruitment of Shank3 from the soluble synaptic pool to the PSD bound pool and might recruit additional Shank2 proteins. Strengthening the PSD scaffold in this way may compensate some deficits seen in forms of ASD caused by imbalance in the Nrxn-Nlg-Shank pathway.

Based on the current data, a model can be proposed according to which zinc supplementation in Shankopathies may be a promising approach. This is based on several observations. First, restoring Shank3 levels in adult mice ameliorates their autism phenotype. Second, increased zinc levels are able to increase synaptic Shank3 levels, which may compensate the loss of one functional copy of the gene. Third, in addition, recruitment of the zinc-dependent Shank2 may contribute to the compensating effect (Figure 2). Given that Shank proteins are found in a complex of further autism associated proteins such as Neurexin (Nrxn) and Neuroligin (Nlgn), as well as mTOR, strengthening Shank3 scaffolds may also be beneficial in cases of mutation of other proteins of the proposed autism associated pathway at excitatory synapses (Bourgeron, 2009).

While PMDS is a very rare syndrome, the prevalence of mutations in Shank3 and Shank2 is higher in the ASD population (~1.5% and 0.17%, respectively, Leblond et al., 2014). In addition, Shank proteins are physiologically linked to proteins with mutations reported in the ASD population such as NMDAR (Pan et al., 2015), Nrxn/Nlgn complexes (Yoo, 2015; Onay et al., 2017) and mGluR5-mTOR (Fragile X syndrome: 1%–2% of patients with ASD, Tuberous sclerosis: ~1% of patients with ASD; Abrahams and Geschwind, 2008). Therefore, this group of patients may benefit from modulation of Shank2/3 via zinc as well.

Besides effects on the Shank2/3 scaffold, activation of zinc signaling may have effects on further synaptic proteins and thereby synapse function (Sensi et al., 2009, 2011). For example, zinc acts as allosteric inhibitor of GluN1/GluN2A (NMDA) receptors (Paoletti et al., 1997). However, it was shown that a postsynaptic increase in zinc can also activate NMDA receptors in a Src tyrosine kinase dependent manner, which may be an important contributor to the rescue of ASD behaviors (Lee et al., 2015). Further, zinc may inhibit GABA_A_ receptors (Smart et al., 1994). Thus, zinc modulates excitatory synaptic transmission as well as inhibitory synaptic transmission and may be an important player in maintaining the balance between excitation (E) and inhibition (I). Changes in E/I ratio have been reported to be important in the pathogenesis ASD (Rubenstein and Merzenich, 2003) and altered zinc homeostasis may positively influence the E/I ratio.

In addition, zinc has inhibitory effects on voltage-gated ion channels (Blakemore and Trombley, 2017), and is linked to BDNF signaling via metalloproteinase activation, which plays an important role in Trk receptor activation (Hwang et al., 2005).

However, with zinc being a non-genetic factor, dosage is important. While genetic models of Shank3 deficiency follow a pattern of full loss (homozygous deletion) or loss of half of Shank3 (heterozygous deletion), zinc will act along a large spectrum of dosages. Finding the correct dosage may be complicated. In addition, it is hard to estimate after which time effects may be observable. While zinc is taken up quickly in the body, it does not cross freely the blood-brain barrier (BBB). A constant elevated serum zinc level may be necessary to generate a sufficient concentration gradient to drive zinc uptake into the brain. Both, dosage and treatment times will be dependent on the type of zinc supplement used. In addition, treatment should be performed as early as possible and therefore, efficacy might critically depend on the ability to diagnose PMDS/ASD early in development. According to previously published data (Grabrucker et al., 2011), forming and immature synapses are characterized by Shank2 and Shank3 family members at the PSD and are more reactive to zinc than mature synapses that also contain the zinc-independent Shank1. Thus, strengthening the Shank2 and Shank3 scaffold to reach a threshold for the synapse to mature will be most critical during the time window of brain development with maximal need for establishment of synaptic contacts to lie down the basic connectivity in and in between brain regions. However, it is possible that some brain regions maintain plasticity throughout later development and will still benefit from increased zinc levels. In line with this, re-expression of Shank3 in adult mice that developed in absence of Shank3 was able to rescue social interaction deficits and repetitive grooming behavior, but not anxiety and motor coordination deficits (Mei et al., 2016). This hints to different time windows in development, where supplementation will be able to rescue different features of ASD to different extent.

Taken together, to move forward in animal studies and finally human studies, various factors need to be considered. The type of supplement needs to ensure effective zinc uptake, but also zinc delivery into the brain. Here, recently developed nanoparticles delivering zinc across the BBB may be promising (Chhabra et al., 2015; Vilella et al., 2017). Additionally, the standard laboratory diet for Shank mouse models has to be carefully controlled for zinc content. Fortified diets used for mice may contain up to five times higher zinc levels as the required daily dosage for mice and it may be possible that heterozygous Shank3 mice on this diet already represent models of low zinc supplementation. While this concentration of zinc may not be enough to cause significant effects, it might contribute to the relatively mild phenotype of heterozygous mice, despite human patients being heterozygous as well. Finally, the age of mice and duration of treatment, as well as dosage of zinc supplementation need to be carefully selected.

Human studies using zinc supplementation in ASD patients so far reported mixed results. While some studies found benefits (Russo and Devito, 2011), the results were less clear in others. In future, the cohort of participants with ASD needs to be more carefully selected based on their underlying genetic mutation, and treatment performed early in life for a long time using therapeutic dosages of classic zinc supplements or alternative forms of zinc supplementation.

## Author Contributions

SH, AKS and AG developed the hypothesis of the study and together drafted the manuscript.

## Conflict of Interest Statement

The authors declare that the research was conducted in the absence of any commercial or financial relationships that could be construed as a potential conflict of interest.
